# 3-[3-(2-Fluoro­benzo­yl)thio­ureido]propionic acid

**DOI:** 10.1107/S1600536814011404

**Published:** 2014-05-24

**Authors:** Nurziana Ngah, Nor Azanita Mohamed, Bohari M. Yamin, Hamizah Mohd Zaki

**Affiliations:** a Kulliyyah of Science, International Islamic University Malaysia, Bandar Indera Mahkota, 25200 Kuantan, Pahang, Malaysia; b School of Chemical Sciences and Food Technology, Faculty of Science and Technology, Universiti Kebangsaan Malaysia, UKM 43600 Bangi Selangor, Malaysia; c Faculty of Applied Sciences, Universiti Teknologi MARA, 40450 Shah Alam, Selangor, Malaysia; d Atta-ur-Rahman Institute for Natural Product Discovery, Universiti Teknologi MARA (UiTM), Puncak Alam Campus, 42300 Bandar Puncak Alam, Selangor D. E., Malaysia

## Abstract

In the title compound, C_10_H_11_FN_3_O_3_S, the 2-fluoro­benzoyl and proponic acid groups maintain a *trans–cis* conformation with respect to the thiono C=S bond across their C—N bonds. The propionic acid group adopts an *anti* conformation about the C—C bond, with an N—C—C—C torsion angle of 173.8 (2)°. The amino groups are involved in the formation of intra­molecular N—H⋯O and N—H⋯F hydrogen bonds. In the crystal, pairs of O—H⋯O hydrogen bonds link mol­ecules into inversion dimers.

## Related literature   

For related structures, see: Yusof *et al.* (2003 ▶); Ngah *et al.* (2006 ▶).
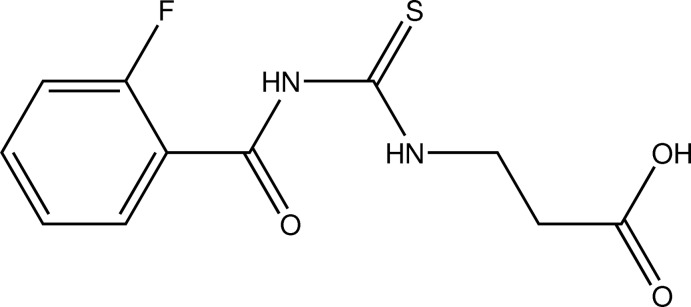


## Experimental   

### 

#### Crystal data  

C_11_H_11_FN_2_O_3_S
*M* *_r_* = 270.28
Monoclinic, 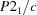

*a* = 11.7103 (7) Å
*b* = 11.1289 (7) Å
*c* = 9.6760 (7) Å
β = 108.407 (2)°
*V* = 1196.49 (14) Å^3^
*Z* = 4
Mo *K*α radiation
μ = 0.29 mm^−1^
*T* = 296 K
0.41 × 0.30 × 0.28 mm

#### Data collection  

Bruker SMART APEX CCD area-detector diffractometer
Absorption correction: multi-scan (*SADABS*; Bruker, 2000 ▶) *T*_min_ = 0.892, *T*_max_ = 0.924
21544 measured reflections
2188 independent reflections
1816 reflections with *I* > 2/s(*I*)
*R*_int_ = 0.032

#### Refinement  

*R*[*F* ^2^ > 2σ(*F* ^2^)] = 0.043
*wR*(*F* ^2^) = 0.134
*S* = 1.13
2188 reflections
167 parameters
1 restraint
H atoms treated by a mixture of independent and constrained refinement
Δρ_max_ = 0.23 e Å^−3^
Δρ_min_ = −0.31 e Å^−3^

### 

Data collection: *SMART* (Bruker, 2000 ▶); cell refinement: *SAINT* (Bruker, 2000 ▶); data reduction: *SAINT*; program(s) used to solve structure: *SHELXS97* (Sheldrick, 2008 ▶); program(s) used to refine structure: *SHELXL97* (Sheldrick, 2008 ▶); molecular graphics: *SHELXTL* (Sheldrick, 2008 ▶); software used to prepare material for publication: *SHELXTL* and *PLATON* (Spek, 2009 ▶).

## Supplementary Material

Crystal structure: contains datablock(s) global, I. DOI: 10.1107/S1600536814011404/cv5458sup1.cif

Structure factors: contains datablock(s) I. DOI: 10.1107/S1600536814011404/cv5458Isup2.hkl

Supporting information file. DOI: 10.1107/S1600536814011404/cv5458Isup3.cml

CCDC reference: 1003660

Additional supporting information:  crystallographic information; 3D view; checkCIF report

## Figures and Tables

**Table 1:** Hydrogen-bond geometry (Å, °)

*D*—H⋯*A*	*D*—H	H⋯*A*	*D*⋯*A*	*D*—H⋯*A*
N1—H1*A*⋯F1	0.86	2.04	2.708 (3)	134
N2—H2*A*⋯O1	0.86	1.97	2.642 (3)	135
O3—H3*A*⋯O2^i^	0.83 (2)	1.82 (2)	2.645 (3)	175 (4)

Symmetry code: (i) 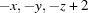.

**Fig. 1.**
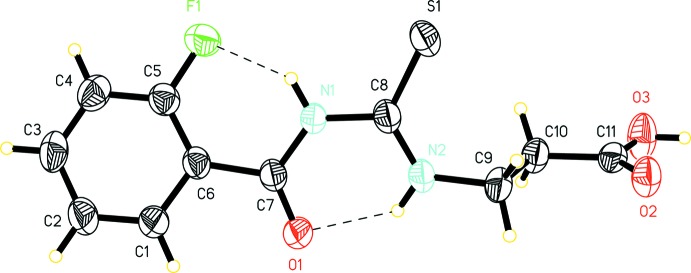
The molecular structure of (I), with displacement ellipsods drawn at the 50% probability level. Dashed lines denote intramolecular hydrogen bonds.

**Fig. 2.**
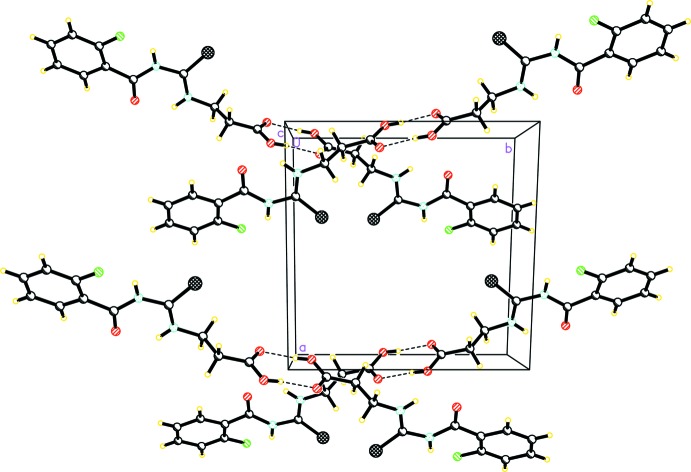
A portion of the molecular packing of (I) viewed down the c axis. Dashed lines denote intermolecular O—H···O hydrogen bonds.

## supplementary crystallographic information

### 1. Comment

In continuation of our study of thiourea derivatives containing propionic acid fragments (Yusof *et al.*, 2003; Ngah *et al.*, 2006), we report here the crystal structure of the title compound (I).

In (I) (Fig.1), all bond lengths and angles are normal and correspond well to those observed in the related compounds (Yusof *et al.*, 2003; Ngah *et al.*, 2006). However, the C11—O3 is slightly shorter [1.306 (3) Å] compared to its analogue [1.325 (4) Å; Ngah *et al.* (2006)] due to electron delocalization along carboxyl group. The molecule maintains its *trans-cis* configuration with respect to the positions of 2-fluorobenzoyl and propionic acid relative to the thino C=S bond across the C8—N1 and C8—N2, respectively. The molecule adopts an *anti* conformation with N2—C9—C10—C11 torsion angle of 173.8 (2)°. In the contrary the analogue adopts a *gauche* conformation with torsion angle of 64.9 (4)°. The 2-fluorophenyl [C1—C6/F1], thiourea [N1/C8/N2/S1] and propionic acid [C9/C10C11/O2/O3] fragments are essentially planar with maximum deviation of 031 (2) Å for atom C10 from the least square plane of propionic acid. The thiourea makes dihedral angles of 20.84 (12)° and 85.78 (11)° with 2-fluorophenyl and propionic acid fragments, respectively. The 2-fluorophenyl is inclined to propionic acid fragments by 65.65 (13)°, compared to 54.29 (19)° in the analogue. There are two intramolecular N1—H1A···F1 and N2—H2A···O1 hydrogen bonds (Table 1) furnishing in the formation of two pseudo six-membered rings (N1—H1A—F1—C5—C6—C7) and (N2—H2A—O1—C7—N1—C8), respectively.

In the crystal structure, the molecules are connected *via* O3—H3A···O2 intermolecular hydrogen bonds to form centrosymmetric dimers (Fig. 2).

### 2. Experimental

30 ml acetone solution of β-alanine (2.92 g, 32.80 mmol) was added into a round-bottom flask containing a solution of 2-fluorobenzoylchloride (5.21 g, 32.80 mmol) and ammonium thiocyanate (2.50 g, 32.80 mmol). The solution mixture was refluxed for 5 h then filtered off into a beaker containing some ice and left to evaporate at room temperature. The yellowish precipitate obtained was washed with water and cold ethanol. The yellowish crytals were obtained by recrystallization of the precipitate in acetonitrile, suitable for X-ray analysis.

### 3. Refinement

The hydroxyl H-atom [O3—H3A] was located from Fourrier map and refined isotropically. Other H atoms were positioned geometrically and refined using riding model with C—H = 0.93–0.97 Å and N—H = 0.86 Å with *U*_iso_(H) = 1.2*U*_eq_(C & N).

### Crystal data

**Table:**

C_11_H_11_FN_2_O_3_S	*F*(000) = 560
*M**_r_* = 270.28	*D*_x_ = 1.500 Mg m^−^^3^
Monoclinic, *P*2_1_/*c*	Mo *K*α radiation, λ = 0.71073 Å
Hall symbol: -P 2ybc	Cell parameters from 13325 reflections
*a* = 11.7103 (7) Å	θ = 2.9–25.5°
*b* = 11.1289 (7) Å	µ = 0.29 mm^−^^1^
*c* = 9.6760 (7) Å	*T* = 296 K
β = 108.407 (2)°	Block, colourless
*V* = 1196.49 (14) Å^3^	0.41 × 0.30 × 0.28 mm
*Z* = 4	

### Data collection

**Table:**

Bruker SMART APEX CCD area-detector diffractometer	2188 independent reflections
Radiation source: fine-focus sealed tube	1816 reflections with *I* > 2/s(*I*)
Graphite monochromator	*R*_int_ = 0.032
Detector resolution: 83.66 pixels mm^-1^	θ_max_ = 25.5°, θ_min_ = 2.9°
ω scan	*h* = −12→14
Absorption correction: multi-scan (*SADABS*; Bruker, 2000)	*k* = −13→13
*T*_min_ = 0.892, *T*_max_ = 0.924	*l* = −11→11
21544 measured reflections	

### Refinement

**Table:**

Refinement on *F*^2^	Primary atom site location: structure-invariant direct methods
Least-squares matrix: full	Secondary atom site location: difference Fourier map
*R*[*F*^2^ > 2σ(*F*^2^)] = 0.043	Hydrogen site location: inferred from neighbouring sites
*wR*(*F*^2^) = 0.134	H atoms treated by a mixture of independent and constrained refinement
*S* = 1.13	*w* = 1/[σ^2^(*F*_o_^2^) + (0.059*P*)^2^ + 0.9312*P*] where *P* = (*F*_o_^2^ + 2*F*_c_^2^)/3
2188 reflections	(Δ/σ)_max_ < 0.001
167 parameters	Δρ_max_ = 0.23 e Å^−^^3^
1 restraint	Δρ_min_ = −0.31 e Å^−^^3^

### Special details

**Table:**

Geometry. All e.s.d.'s (except the e.s.d. in the dihedral angle between two l.s. planes) are estimated using the full covariance matrix. The cell e.s.d.'s are taken into account individually in the estimation of e.s.d.'s in distances, angles and torsion angles; correlations between e.s.d.'s in cell parameters are only used when they are defined by crystal symmetry. An approximate (isotropic) treatment of cell e.s.d.'s is used for estimating e.s.d.'s involving l.s. planes.
Refinement. Refinement of *F*^2^ against ALL reflections. The weighted *R*-factor *wR* and goodness of fit *S* are based on *F*^2^, conventional *R*-factors *R* are based on *F*, with *F* set to zero for negative *F*^2^. The threshold expression of *F*^2^ > σ(*F*^2^) is used only for calculating *R*-factors(gt) *etc*. and is not relevant to the choice of reflections for refinement. *R*-factors based on *F*^2^ are statistically about twice as large as those based on *F*, and *R*- factors based on ALL data will be even larger.

### Fractional atomic coordinates and isotropic or equivalent isotropic displacement parameters (Å^2^)

**Table:**

	*x*	*y*	*z*	*U*_iso_*/*U*_eq_	
F1	0.40942 (16)	0.72653 (14)	0.75758 (18)	0.0567 (5)	
S1	0.36160 (7)	0.36649 (6)	0.92829 (10)	0.0596 (3)	
O1	0.16343 (16)	0.70323 (15)	0.9833 (2)	0.0502 (5)	
O2	0.07405 (16)	0.12040 (15)	1.06970 (18)	0.0453 (4)	
O3	−0.0317 (2)	0.09343 (17)	0.8376 (2)	0.0585 (6)	
N1	0.30302 (18)	0.59538 (16)	0.9185 (2)	0.0398 (5)	
H1A	0.3627	0.6022	0.8853	0.048*	
N2	0.17782 (18)	0.46625 (17)	0.9899 (2)	0.0411 (5)	
H2A	0.1364	0.5295	0.9928	0.049*	
C1	0.2683 (2)	0.9209 (2)	0.9548 (3)	0.0471 (6)	
H1	0.2171	0.9175	1.0112	0.056*	
C2	0.3106 (3)	1.0305 (2)	0.9274 (4)	0.0548 (7)	
H2	0.2883	1.1001	0.9656	0.066*	
C3	0.3859 (2)	1.0375 (2)	0.8437 (3)	0.0501 (7)	
H3	0.4143	1.1118	0.8250	0.060*	
C4	0.4193 (2)	0.9348 (2)	0.7879 (3)	0.0476 (6)	
H4	0.4704	0.9388	0.7314	0.057*	
C5	0.3761 (2)	0.8260 (2)	0.8168 (3)	0.0388 (5)	
C6	0.3002 (2)	0.8147 (2)	0.9001 (3)	0.0359 (5)	
C7	0.2487 (2)	0.7005 (2)	0.9367 (3)	0.0367 (5)	
C8	0.2736 (2)	0.4784 (2)	0.9472 (3)	0.0385 (5)	
C9	0.1380 (2)	0.3521 (2)	1.0327 (3)	0.0417 (6)	
H9A	0.0899	0.3673	1.0960	0.050*	
H9B	0.2079	0.3057	1.0873	0.050*	
C10	0.0648 (2)	0.2795 (2)	0.9026 (3)	0.0414 (6)	
H10A	−0.0095	0.3218	0.8541	0.050*	
H10B	0.1095	0.2716	0.8341	0.050*	
C11	0.0357 (2)	0.1574 (2)	0.9458 (3)	0.0363 (5)	
H3A	−0.041 (3)	0.0267 (17)	0.871 (4)	0.079 (11)*	

### Atomic displacement parameters (Å^2^)

**Table:**

	*U*^11^	*U*^22^	*U*^33^	*U*^12^	*U*^13^	*U*^23^
F1	0.0767 (11)	0.0438 (9)	0.0639 (10)	0.0036 (7)	0.0428 (9)	−0.0009 (7)
S1	0.0532 (4)	0.0281 (3)	0.1082 (7)	0.0000 (3)	0.0408 (4)	−0.0041 (3)
O1	0.0457 (10)	0.0338 (9)	0.0824 (14)	−0.0014 (7)	0.0365 (10)	−0.0019 (8)
O2	0.0572 (11)	0.0356 (9)	0.0410 (10)	−0.0121 (8)	0.0124 (8)	0.0000 (7)
O3	0.0773 (14)	0.0440 (11)	0.0450 (11)	−0.0249 (10)	0.0063 (9)	0.0003 (9)
N1	0.0378 (11)	0.0265 (9)	0.0618 (13)	−0.0024 (8)	0.0253 (10)	0.0007 (9)
N2	0.0407 (11)	0.0271 (10)	0.0597 (13)	−0.0051 (8)	0.0217 (10)	−0.0010 (9)
C1	0.0442 (14)	0.0329 (12)	0.0716 (18)	0.0012 (10)	0.0290 (13)	−0.0010 (12)
C2	0.0538 (16)	0.0296 (13)	0.085 (2)	0.0008 (11)	0.0277 (15)	−0.0019 (13)
C3	0.0485 (15)	0.0344 (13)	0.0655 (17)	−0.0057 (11)	0.0154 (13)	0.0111 (12)
C4	0.0499 (15)	0.0473 (14)	0.0492 (15)	−0.0023 (11)	0.0205 (12)	0.0103 (12)
C5	0.0429 (13)	0.0344 (12)	0.0395 (12)	0.0026 (10)	0.0132 (10)	0.0029 (10)
C6	0.0341 (12)	0.0275 (11)	0.0452 (13)	0.0013 (9)	0.0111 (10)	0.0027 (9)
C7	0.0358 (12)	0.0275 (11)	0.0472 (13)	−0.0017 (9)	0.0138 (10)	−0.0004 (9)
C8	0.0382 (12)	0.0280 (11)	0.0498 (14)	−0.0040 (9)	0.0143 (11)	−0.0021 (10)
C9	0.0471 (14)	0.0331 (12)	0.0483 (14)	−0.0098 (10)	0.0200 (11)	0.0000 (10)
C10	0.0452 (14)	0.0359 (12)	0.0441 (13)	−0.0097 (10)	0.0156 (11)	0.0013 (10)
C11	0.0362 (12)	0.0347 (12)	0.0404 (13)	−0.0060 (9)	0.0156 (10)	−0.0031 (10)

### Geometric parameters (Å, º)

**Table:**

F1—C5	1.360 (3)	C2—C3	1.375 (4)
S1—C8	1.663 (2)	C2—H2	0.9300
O1—C7	1.219 (3)	C3—C4	1.372 (4)
O2—C11	1.212 (3)	C3—H3	0.9300
O3—C11	1.306 (3)	C4—C5	1.375 (3)
O3—H3A	0.830 (10)	C4—H4	0.9300
N1—C7	1.369 (3)	C5—C6	1.380 (3)
N1—C8	1.397 (3)	C6—C7	1.497 (3)
N1—H1A	0.8600	C9—C10	1.514 (3)
N2—C8	1.319 (3)	C9—H9A	0.9700
N2—C9	1.458 (3)	C9—H9B	0.9700
N2—H2A	0.8600	C10—C11	1.491 (3)
C1—C2	1.373 (4)	C10—H10A	0.9700
C1—C6	1.393 (3)	C10—H10B	0.9700
C1—H1	0.9300		
			
C11—O3—H3A	107 (3)	C5—C6—C7	126.7 (2)
C7—N1—C8	128.1 (2)	C1—C6—C7	117.0 (2)
C7—N1—H1A	115.9	O1—C7—N1	122.5 (2)
C8—N1—H1A	115.9	O1—C7—C6	120.3 (2)
C8—N2—C9	123.9 (2)	N1—C7—C6	117.2 (2)
C8—N2—H2A	118.0	N2—C8—N1	116.4 (2)
C9—N2—H2A	118.0	N2—C8—S1	125.12 (18)
C2—C1—C6	121.6 (2)	N1—C8—S1	118.43 (17)
C2—C1—H1	119.2	N2—C9—C10	112.2 (2)
C6—C1—H1	119.2	N2—C9—H9A	109.2
C1—C2—C3	120.1 (2)	C10—C9—H9A	109.2
C1—C2—H2	120.0	N2—C9—H9B	109.2
C3—C2—H2	120.0	C10—C9—H9B	109.2
C4—C3—C2	120.0 (2)	H9A—C9—H9B	107.9
C4—C3—H3	120.0	C11—C10—C9	111.9 (2)
C2—C3—H3	120.0	C11—C10—H10A	109.2
C3—C4—C5	118.9 (2)	C9—C10—H10A	109.2
C3—C4—H4	120.5	C11—C10—H10B	109.2
C5—C4—H4	120.5	C9—C10—H10B	109.2
F1—C5—C4	117.3 (2)	H10A—C10—H10B	107.9
F1—C5—C6	119.7 (2)	O2—C11—O3	123.3 (2)
C4—C5—C6	123.0 (2)	O2—C11—C10	122.7 (2)
C5—C6—C1	116.3 (2)	O3—C11—C10	114.0 (2)
			
C6—C1—C2—C3	0.3 (4)	C5—C6—C7—O1	−163.9 (2)
C1—C2—C3—C4	−0.3 (4)	C1—C6—C7—O1	15.8 (4)
C2—C3—C4—C5	0.2 (4)	C5—C6—C7—N1	17.5 (4)
C3—C4—C5—F1	178.9 (2)	C1—C6—C7—N1	−162.8 (2)
C3—C4—C5—C6	−0.1 (4)	C9—N2—C8—N1	−176.3 (2)
F1—C5—C6—C1	−178.8 (2)	C9—N2—C8—S1	2.6 (4)
C4—C5—C6—C1	0.1 (4)	C7—N1—C8—N2	3.9 (4)
F1—C5—C6—C7	0.9 (4)	C7—N1—C8—S1	−175.0 (2)
C4—C5—C6—C7	179.8 (2)	C8—N2—C9—C10	−82.4 (3)
C2—C1—C6—C5	−0.2 (4)	N2—C9—C10—C11	173.8 (2)
C2—C1—C6—C7	−180.0 (2)	C9—C10—C11—O2	−4.5 (3)
C8—N1—C7—O1	0.2 (4)	C9—C10—C11—O3	177.3 (2)
C8—N1—C7—C6	178.8 (2)		

### Hydrogen-bond geometry (Å, º)

**Table:**

*D*—H···*A*	*D*—H	H···*A*	*D*···*A*	*D*—H···*A*
N1—H1*A*···F1	0.86	2.04	2.708 (3)	134
N2—H2*A*···O1	0.86	1.97	2.642 (3)	135
O3—H3*A*···O2^i^	0.83 (2)	1.82 (2)	2.645 (3)	175 (4)

Symmetry code: (i) −*x*, −*y*, −*z*+2.
